# *Saccharomyces boulardii* as a probiotic yeast in food applications and its health properties: a review and future perspectives

**DOI:** 10.3389/fnut.2026.1864466

**Published:** 2026-07-08

**Authors:** Zijie Dong, Longfei Wang, Yongqiang An, Tianyi Wang, Hao Dong, Qiuxiang Li, Hao Li, Lishui Chen

**Affiliations:** 1 College of Agriculture, Henan University, Kaifeng, China; 2 Food Laboratory of Zhongyuan, Luohe, China; 3 The School of Food Science and Technology, Harbin Institute of Technology, Harbin, China; 4 School of Food Science and Technology, Henan University of Technology, Zhengzhou, China; 5 College of Food and Bioengineering, Zhengzhou University of Light Industry, Zhengzhou, China

**Keywords:** fermented foods, gastrointestinal disorders, gut microbiota, mechanisms of action, probiotic yeast, *Saccharomyces boulardii*

## Abstract

*Saccharomyces boulardii* is currently the only probiotic yeast with established clinical validation for use in both humans and livestock. It exhibits unique advantages that are difficult to achieve with conventional probiotics, including resistance to gastric acid and antibiotics, as well as broad-spectrum antimicrobial activity. Moreover, through the secretion of bioactive metabolites, *S. boulardii* can improve food quality and contribute to the management of digestive disorders. Consequently, it has gained increasing attention in functional ingredients (FI), fermented foods (FF), and foods for special medical purposes (FSMP). This review involves reviewing and screening relevant scientific literature to conduct a critical bibliometric analysis. This review provides a systematic review of the physiological characteristics, mechanisms of action, applications in food systems, application scope, and clinically validated health benefits of *S. boulardii*. These benefits include modulation of the gut microbiota, enhancement of immune function, and alleviation of symptoms associated with gastrointestinal disorders. The review also covers advances in genetic engineering approaches aimed at enhancing its probiotic functions, as well as the technical, regulatory, and safety limitations associated with its application in food products. Finally, in light of current bottlenecks associated with its industrial application, future research directions are proposed.

## 1 Introduction

Changes in modern lifestyles, characterized by diets high in sugar and fat and low in fiber, along with the widespread use of antibiotics, have contributed to increasing imbalances in the human gut microbiota, consequently triggering a range of gastrointestinal disorders, including diarrhea, inflammatory bowel disease, and irritable bowel syndrome (1, 2). Probiotics have emerged as an important strategy for regulating gut microbiota and maintaining intestinal health, and have received considerable attention in recent years (3). The Food and Agriculture Organization of the United Nations (FAO) and the World Health Organization (WHO) define probiotics as “live microorganisms which, when administered in adequate amounts, confer a health benefit on the host” (4). In recent years, the global probiotics market has expanded rapidly. Sales of probiotic foods and dietary supplements reached USD 54.77 billion in 2020. The market is projected to achieve a compound annual growth rate (CAGR) of 7.5% from 2021 to 2030, exceeding USD 96 billion by 2030, of which fungus-derived probiotic products are expected to exhibit a CAGR of 12.4%, significantly higher than that of bacterial sources (5, 6). Traditional probiotic research has primarily focused on bacterial genera such as *Lactobacillus* and *Bifidobacterium*. However, as probiotic research has progressed, yeasts, particularly *Saccharomyces boulardii*, have become an important focus in both research and application due to their distinctive physiological characteristics and beneficial functions.

*S. boulardii* was first isolated by the French microbiologist Henri Boulard in 1920 from the peels of lychee and mangosteen fruits in Southeast Asia. It is the only yeast strain with well-established probiotic properties identified to date and is currently classified as a variant of *Saccharomyces cerevisiae* (7). Compared with bacterial probiotics (e.g., *Lactobacillus* and *Bifidobacterium*), *S. boulardii* offers distinct advantages: (i) higher resistance to gastric acid and bile salts, ensuring better survival through the gastrointestinal tract; (ii) intrinsic tolerance to multiple antibiotics, allowing co-administration during antibiotic therapy; and (iii) secretion of unique antimicrobial metabolites such as phenyllactic acid and 2-hydroxyisocaproic acid. These features make *S. boulardii* a particularly promising probiotic for both food applications and clinical adjunct therapy (8, 9). Furthermore, unlike many other yeast species, *S. boulardii* has an optimal growth temperature of approximately 37 °C, enabling it to function effectively at human body temperature and supporting its use in oral probiotic formulations (10).

In recent years, the application of *S. boulardii* in the food industry has gradually increased, demonstrating good adaptability and stability across a range of products, particularly in fermented dairy products (11), fruit juices (12), beer (13), functional beverages and FSMP (14). While FSMP refers to foods specifically formulated for those with restricted intake, malabsorption, or metabolic disorders, they should be recognized strictly as nutritional support and not as substitutes for pharmaceutical treatment. In addition to enhancing flavor and texture, *S. boulardii* improves the functional properties of food products by promoting the growth of lactic acid bacteria and modulating the fermentation environment. For instance, incorporating *S. boulardii* into yogurt has been shown to enhance its antioxidant activity, extend shelf life, and improve sensory qualities (15). In products such as ice cream and cheese, it remains viable under adverse conditions, including low temperatures and high osmotic pressure, making it an ideal probiotic carrier (16). In fermented beverages such as barley wort, *S. boulardii* can significantly increase the production of oligosaccharides, thereby enhancing their bioavailability, particularly for individuals with suboptimal health status (17). Additionally, *S. boulardii* can be incorporated into microencapsulation techniques using materials such as alginate and fruit pulp to develop edible bioactive films for food preservation (18). Furthermore, postbiotics derived from *S. boulardii* fermentation have been used to produce coatings that extend the shelf life of lamb meat (19).

More importantly, *S. boulardii* exhibits significant health benefits, including the regulation of gut microbiota, enhancement of immune function, and inhibition of pathogenic microorganisms. Numerous *in vitro* and *in vivo* studies have demonstrated that *S. boulardii* exerts its probiotic effects through multiple mechanisms. These mechanisms include the inhibition of adhesion and colonization of pathogenic bacteria, such as *Escherichia coli*, *Salmonella*, and *Clostridioides difficile* (20). In addition, *S. boulardii* can degrade bacterial toxins, for example, by inhibiting the activity of *C. difficile* toxin A (21). It also enhances intestinal barrier function by suppressing the TLR2/MYD88/NF-κB signaling pathway, thereby maintaining epithelial integrity (22). Furthermore, *S. boulardii* modulates host immune responses by promoting the expression of cytokines, including interleukin-8 (IL-8), IL-10, and IL-1β, as well as antioxidant enzymes such as catalase (CAT), glutathione peroxidase (Gpx), and superoxide dismutase (SOD) (9, 23). It also produces metabolites with antimicrobial activity, including organic acids such as phenyllactic acid and 2-hydroxyisocaproic acid, which have been detected at increased levels in the polysaccharide mucilage of *S. boulardii* ATCC MYA-796 (19). Furthermore, *S. boulardii* has been associated with other functional effects, including antioxidant activity, cholesterol reduction, and the alleviation of lactose intolerance (12, 24, 25).

Although *S. boulardii* shows considerable potential for applications in the food and health sectors, further research is required to assess its stability across different food matrices, its interactions with commensal microbiota, and the safety of long-term consumption. Furthermore, advances in synthetic biology and genetic engineering have enabled the development of engineered *S. boulardii* strains with enhanced therapeutic functionality. An increasing number of studies have employed gene-editing tools, CRISPR/Cas9, to modify *S. boulardii* for targeted applications (26), such as expressing vaccine antigens (27), anti-inflammatory factors (28), and antimicrobial peptides (29), thereby expanding their potential application in disease prevention and treatment.

*S. boulardii*, as a probiotic with excellent physiological characteristics and a wide range of health benefits, holds considerable promise for application in the development of functional foods and health intervention strategies. The incorporation of probiotic yeasts into food systems as bioactive components with both health-promoting and therapeutic effects represents an emerging and promising strategy to enhance the functional value of foods. This review uses a mechanism-matrix-evidence-translation model: molecular effectors are first identified; their behavior is then evaluated under food-processing variables; product performance is quantified using viability, metabolite yield, sensory acceptability, and shelf-life endpoints; and health claims are interpreted through RCT evidence, risk of bias, GRADE certainty, and food-matrix directness. This review article systematically summarizes the probiotic properties of *S. boulardii*, its applications in food systems, and the current challenges associated with its use, aiming to provide a comprehensive reference to support further in-depth research and facilitate its broader application in the food industry and human health.

## 2 Literature search and data collection

### 2.1 Review type and PICOS framework

This review is conducted as a systematic review following the PRISMA (Preferred Reporting Items for Systematic Reviews and Meta-Analyses) guidelines. The following PICOS framework was predefined to guide the literature search and selection:

Population (P): Humans, animals, or *in vitro* models relevant to gastrointestinal health or food applications.

Intervention (I): Administration of live *S. boulardii* (any strain, single or combined with other probiotics) in a food matrix (e.g., fermented dairy, plant-based products, beverages, baked goods, edible films) or as a dietary supplement with clear food context.

Comparison (C): Placebo, no treatment, conventional probiotics (e.g., *Lactobacillus*, *Bifidobacterium*), or non-probiotic controls.

Outcome (O): Modulation of gut microbiota, immune function, gastrointestinal symptoms (diarrhea, IBS, IBD), antimicrobial activity, food quality/sensory properties, or probiotic stability/survival in food.

Study design (S): Original research articles, clinical trials (randomized or non-randomized), observational studies (cohort, cross-sectional), *in vitro* and animal studies that provide mechanistic insights directly linked to human health or food applications.

### 2.2 Search strategy and search terms

This review was conducted across major scientific databases such as Google scholar, Science Direct, PubMed, Scopus, Embase and Web of Science following PRISMA guidelines (30). The search period ranged from the inception of each database to March 1, 2026. The search strategy combined terms related to the microorganism, its functional and food-related applications, and its health effects. The following key words and Boolean operators were used: (“*Saccharomyces boulardii*” OR “*S. boulardii*” OR “probiotic yeast”) AND (“fermented foods” OR “functional ingredients” OR “FSMP” OR “food matrix”) AND (“gut microbiota” OR “gastrointestinal disorders” OR “immune modulation” OR “antimicrobial activity”) AND (“clinical trial” OR “health benefit” OR “safety” OR “genetic engineering”). Additional filters were applied to include only original research articles, clinical trials, observational studies, and systematic reviews written in English. This language restriction was implemented due to limitations in reliable translation and to ensure methodological consistency.

Two independent researchers performed literature searches in each database.

### 2.3 Inclusion and exclusion criteria

The research articles initially included in the analysis were all published by January 31, 2026. A subsequent search was conducted to update the analysis through March 1, 2026. We excluded duplicate records identified across databases, review articles (including systematic reviews), conference proceedings, as well as any articles published in languages other than English. The eligible references in the reference list were then screened and selected for analysis based on the following inclusion and exclusion criteria:

#### 2.3.1 Inclusion criteria

Studies focusing on *S. boulardii* (including its subspecies or commercial strains) regarding its physiological characteristics, mechanisms of action, applications in food systems (functional ingredients, fermented foods, foods for special medical purposes), and clinically validated health benefits.

Original research articles, clinical trials, observational studies (cohort, cross-sectional), and systematic reviews with or without meta-analysis.

Studies reporting outcomes related to gut microbiota modulation, immune function enhancement, alleviation of gastrointestinal disorders, improvement of food quality, or genetic engineering of *S. boulardii*.

Studies where *S. boulardii* is administered as live yeast cells within a food matrix (e.g., fermented products, functional foods) or as a probiotic supplement, provided the food matrix is clearly described.

Human studies (intervention or observational) as well as *in vitro* and animal studies that provide mechanistic insights relevant to human health or food applications, when explicitly linked to S*. boulardii*.

#### 2.3.2 Exclusion criteria

Studies focusing on probiotic yeasts other than *S. boulardii* (e.g., *S. cerevisiae* var. *boulardii* is included only if explicitly stated as *S. boulardii*; other species such as *Kluyveromyces*, *Pichia*, or non- *S. boulardii* strains are excluded).

Use of heat-killed, UV-inactivated, or non-viable *S. boulardii* preparations, unless the study explicitly compares live vs. inactivated forms and the main focus remains on live yeast.

Studies that only investigate chemical, physicochemical, or sensory properties of foods without any microbiological or health-related outcome.

Supplementation with *S. boulardii* carried by a non-food matrix (e.g., encapsulated pharmaceutical products) without a clear food application context.

Animal or cellular studies that do not provide translatable insights into human gut microbiota, food fermentation, or clinical health effects (purely basic mechanistic studies without any link to food or human health are excluded).

Reviews, congress proceedings, editorials, letters, commentaries, or duplicate publications without original data.

Articles written in languages other than English.

Studies where the gut microbiota or health outcome is assessed exclusively by indirect methods (e.g., only by culture-based enumeration without molecular confirmation, or solely by detection of microbial metabolites) unless they provide significant evidence of colonization or health benefit.

### 2.4 Data extraction

The data was collected in a standardized format using Excel and recorded as follows:

Bibliographic information (author, year, country).

*S. boulardii* used in food products was investigated.

Specific components of the fermentation strain.

Beneficial effects on humans and animals.

Experimental models and methods.

Specific scope of application.

### 2.5 Quality assessment, risk of bias and GRADE certainty

The methodological quality of included studies was evaluated using study-type-specific tools. Randomized trials were assessed with Cochrane RoB 2; non-randomized human studies with ROBINS-I; animal studies with SYRCLE; and food-matrix studies with a bespoke technological-quality checklist covering strain authentication, inoculum level, enumeration method, matrix composition, thermal/non-thermal processing conditions, storage temperature and duration, sensory design, and statistical reporting. Two reviewers independently completed the assessments and resolved disagreements by consensus.

A GRADE-based certainty assessment was added for the main clinical and food-technology outcome. Certainty was downgraded for high or unclear risk of bias, inconsistency between trials, indirectness caused by supplement rather than food delivery, imprecision, and suspected publication bias; it was upgraded only when there was a plausible dose–response relationship, a large consistent effect, or strong mechanistic support that was directly linked to a food matrix. Thus, clinical outcomes were not treated as equivalent unless the intervention, strain, dose, population, and delivery matrix were comparable.

For food applications, evidence certainty was reported separately from clinical certainty. For example, a matrix may show high technological stability but low clinical directness if no human trial used that food vehicle. Conversely, a clinical capsule trial may support strain efficacy but was downgraded for food-application claims if viability, release kinetics, sensory acceptability, and processing losses were not measured in the final food product.

## 3 Results

### 3.1 Literature search and data extraction

**Figure 1:**
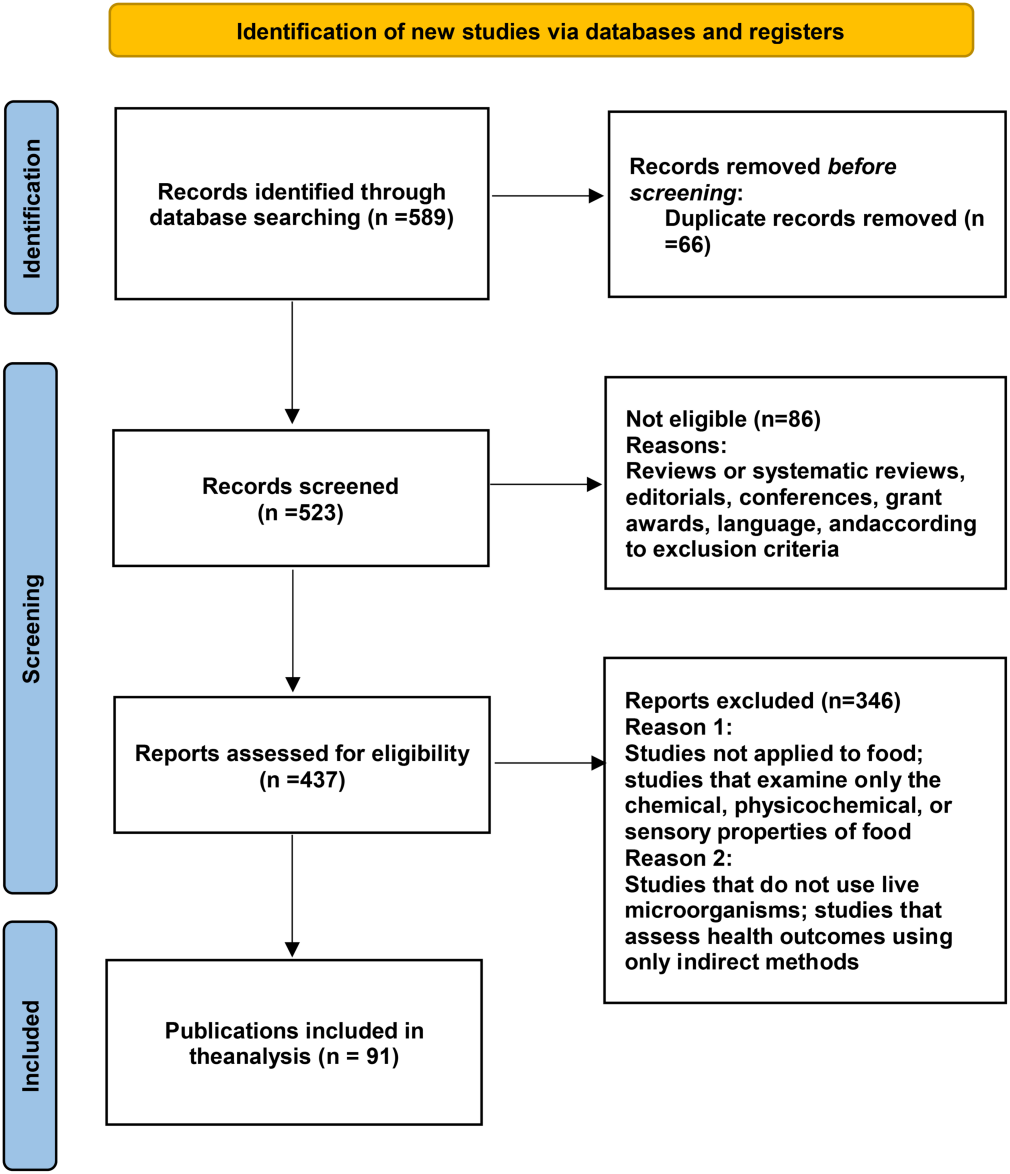
The PRISMA flowchart illustrates the complete literature search and data compilation process used in the review of the applications of *S. boulardii* in fermented foods.

The PRISMA framework was applied systematically through three stages: identification, screening and included. During the identification stage, the databases Google scholar, Science Direct, and Web of Science were searched independently using the predefined search strategy (see Section 2.1). The initial search yielded a total of 589 records. After removing 66 duplicate records, 523 articles proceeded to the screening stage for article type and language. Two independent reviewers screened the papers based on inclusion criteria regarding article type and language, ultimately excluding 86 records; the remaining 437 full-text articles were assessed for eligibility. During this stage, 346 articles were excluded for the following reasons: use of heat-killed or non-viable *S. boulardii* without comparison to live cells (*n =* 61), exclusive focus on chemical/sensory properties without health-related outcomes (*n =* 65), non-food matrix (e.g., encapsulated pharmaceutical products without food context) (*n =* 121), Health assessments conducted solely through indirect methods (*n =* 75) and other reasons for failing to meet the screening criteria (*n =* 24). Finally, a total of 91 peer-reviewed studies met all inclusion criteria and were included in the qualitative synthesis. The detailed numbers of records at each stage are shown in the flow diagram (Figure 1).

## 4 Discussion

### 4.1 Mechanism-matrix-evidence translation framework

To integrate the applications of *S. boulardii* in food and clinical settings, this review is organized around a five-level framework. Level 1 identifies molecular effectors, including toxin-degrading enzymes, *β*-glucan/mannan receptor ligands, aromatic amino acid metabolites, and engineered therapeutic payloads. Level 2 evaluates how food processing controls those effectors through pH, temperature, oxygen/redox state, available sugars and amino acids, phenolics, water activity, and storage. Level 3 quantifies product performance using viable counts, metabolite concentration, LAB synergy, texture, volatile profile, and consumer acceptability. Level 4 grades the health evidence and distinguishes direct food-matrix RCTs from supplement trials. Level 5 examines scalability, regulatory status, quality-by-design controls, and biosafety. Subsequent sections explicitly map evidence to this framework.

### 4.2 Strain characteristics and mechanisms of action of *S. boulardii*

#### 4.2.1 Physiological and genetic characteristics

*S. boulardii* is a variant of *S. cerevisiae*. This probiotic exhibits a degree of antibiotic tolerance and can rapidly utilize carbohydrates, fermenting them to produce specific metabolites (31). Its optimal growth temperature ranges from approximately 30 to 37 °C, enabling robust growth at human body temperature, making it suitable for use as an oral probiotic. Its cell wall is rich in β-1,3-glucan and mannoproteins, enabling it to maintain more than 90% viability under conditions of pH 2.0 and 0.3% bile salts (32). Compared with *S. cerevisiae* and other yeasts, *S. boulardii* additionally carries gene clusters encoding indole-3-lactic acid, phenyllactic acid, and polyketide synthase (PKS), which are associated with enhanced antimicrobial and anti-inflammatory potential (33).

### 4.3 Mechanisms of action in the intestinal tract

#### 4.3.1 Antimicrobial activity and toxin reduction

**Figure 2:**
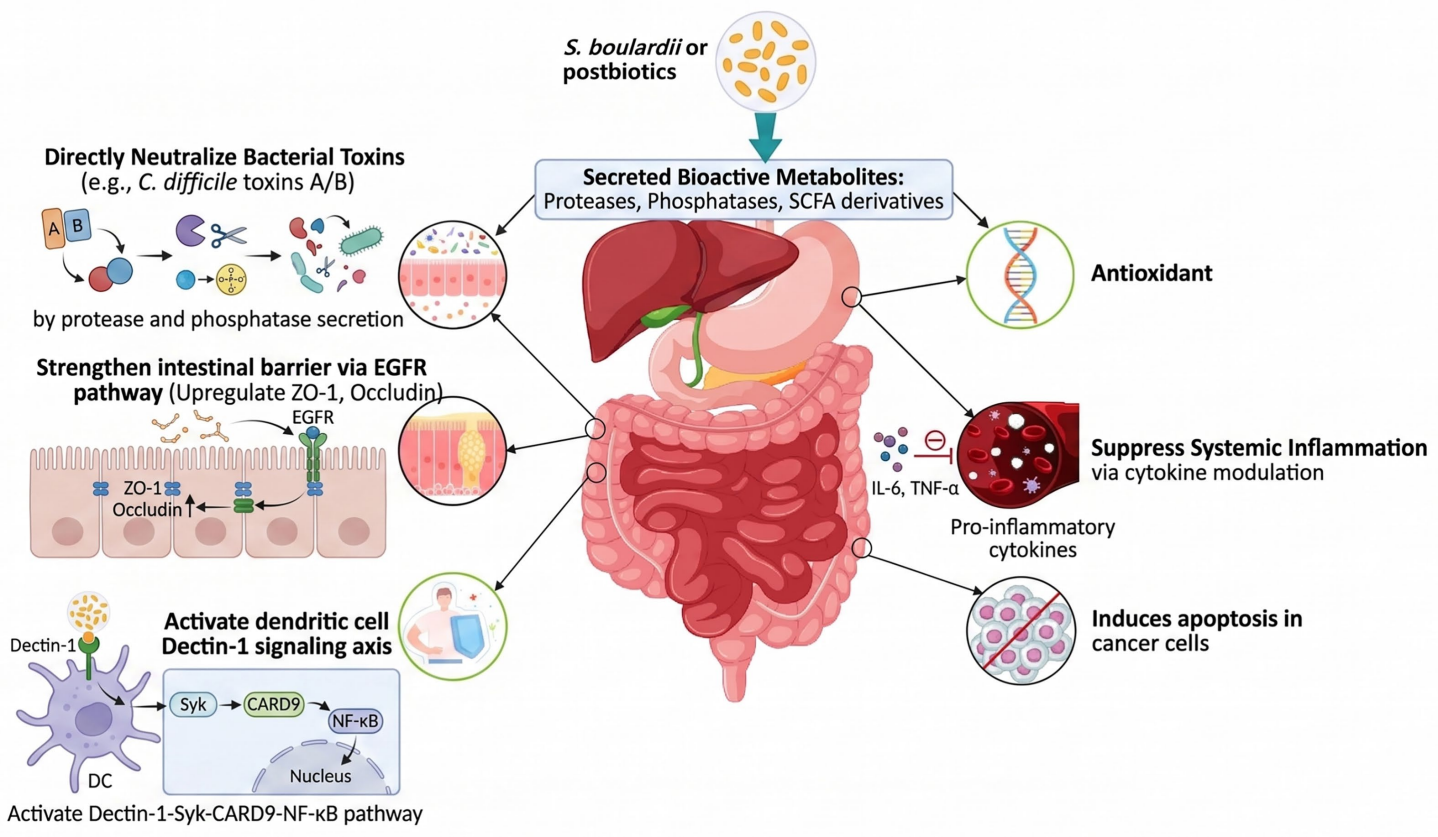
Therapeutic function of *S. boulardii* in the human digestive system. *S. boulardii* acts through a multifaceted pathway involving the secretion of bioactive metabolites. These components **(A)** directly neutralize bacterial toxins; **(B)** activate the Dectin-1-Syk-CARD9-NF-κB signaling axis to modulate dendritic cell responses; and **(C)** strengthen the intestinal barrier by upregulating tight junction protein (ZO-1, occludin) expression via the epidermal growth factor receptor (EGFR) signaling pathway.

*S. boulardii* generally exerts its effects in the human intestinal tract either as live cells or through secreted metabolites, with the common pathways and associated functions shown in Figure 2. Studies have demonstrated that *S. boulardii* achieves a dual “toxin–bacteria” elimination effect through the secretion of highly active proteases and phosphatases (34, 35). *S. boulardii* exerts antimicrobial activity through separable but interacting mechanisms. First, live cells secrete a 54-kDa serine protease that hydrolyzes receptor-binding regions of *C. difficile* toxins A and B, thereby reducing epithelial binding, neutrophil recruitment, and toxin-driven inflammation (36). Second, a 120-kDa phosphatase dephosphorylates bacterial LPS and reduces toxin-induced cAMP accumulation and IL-8 release in epithelial models (37). Third, fermentation metabolites such as capric acid, phenyllactic acid, 2-hydroxyisocaproic acid, and peptide fractions lower pathogen fitness through membrane disruption, intracellular acidification, and interference with adhesion (38, 39). The relative importance of these routes is matrix-dependent: live chilled dairy systems can maintain enzyme secretion, whereas baked or extruded foods are more likely to deliver paraprobiotic cell-wall ligands or heat-stable organic acids.

#### 4.3.2 Intestinal barrier repair and mucus homeostasis

Both *in vitro* and *in vivo* studies have consistently confirmed that live or heat-inactivated *S. boulardii* cells can restore epithelial integrity by upregulating MUC2 gene transcription and increasing the expression of tight junction proteins, including ZO-1, occludin, and claudin-1 (40, 41). In a dextran sulphate sodium(DSS)-induced colitis mouse model, oral administration of *S. boulardii* CNCM I-745 for 7 days resulted in a 2.1-fold increase in colonic MUC2 mRNA levels and a significant enhancement of ZO-1 fluorescence intensity, along with a 34% decrease in the lactulose/mannitol (L/M) ratio, indicating improved intestinal barrier function and reduced inflammation (42). This effect has been associated with the activation of the *β*-glucan-Dectin-1-Akt signaling pathway. Moreover, thioredoxin (Trx), a metabolite of *S. boulardii*, has been shown to effectively promote the expression of EGFR ligand genes and to transactivate EGFR in a concentration-dependent manner (43).

#### 4.3.3 Immunomodulation and inflammation suppression

Studies have shown that *S. boulardii* regulates intestinal mucosal immunity through two main pathways: direct yeast-immune cell interactions and metabolite-receptor signaling (44, 45). *S. boulardii exerts* anti-inflammatory and immunomodulatory effects by inhibiting nuclear factor (NF)-κB activation, IL-8 gene expression, and IL-8 production, as well as reducing inflammatory signaling in both lymphocytes and non-lymphocytes. It also suppresses the activation of human dendritic cells (DCs) and LPS-induced T-cell proliferation (22). On one hand, β-glucan in the cell wall of *S. boulardii* is recognized by Dectin-1 on DCs, activating the Syk-CARD9-NF-κB signaling pathway and promoting IL-10 production and Treg differentiation while inhibiting Th17 polarization. On the other hand, small peptides secreted by the yeast can block LPS-TLR4 binding, downregulate downstream MAPK signaling and NF-κB p65 phosphorylation, and significantly reduce levels of IL-8, TNF-*α*, and IL-1β (46). In an antibiotic-induced dysbiosis model, *S. boulardii* has been shown to accelerate microbiota reconstruction by reducing CD80 and CD197 inflammatory DCs and blocking excessive T-cell activation (47). This duality explains why live, heat-inactivated, and fragmented cells can all be bioactive but not functionally identical. Processing that exposes β-glucan epitopes may enhance PRR recognition, whereas severe Maillard reaction, oxidation, or aggregation may mask ligands and reduce activity.

#### 4.3.4 Anticancer activity and maintenance of gut homeostasis

Yeast postbiotics are defined as well-characterized, inactivated or purified non-viable yeast cells, together with their cell wall polysaccharides and fermentation metabolites, whose biological functions have been validated. These non-viable preparations can provide benefits comparable to live probiotics and serve as “non-live functional ingredients” that combine safety with multiple biological activities. Recent studies have demonstrated that *S. boulardii*-derived postbiotics, including heat-inactivated cells, cell wall fragments, and fermentation supernatants, retain significant biological activity (48). In Caco-2 cells treated with *S. boulardii* metabolites, the relative expression of survivin, IL-8, and NFκB was significantly reduced, accompanied by induction of apoptosis in these human colon cancer cells. After 24 h of treatment with the metabolites, the apoptosis rate of Caco-2 cells reached 62.23% (49). In addition, indole-3-lactic acid, phenyllactic acid, and purine nucleosides present in the fermentation supernatant can upregulate the expression of SOD-1 and quinone reductase (QR) genes through activation of the Nrf2/ARE signaling pathway (50).

### 4.4 Application forms of *S. boulardii* in food

#### 4.4.1 Live cell preparations

*S. boulardii* is one of the most widely used live-cell probiotic forms (11). The primary advantage of live *S. boulardii* lies in its capacity for real-time metabolic activity and transient intestinal colonization. At the molecular level, live cells actively secrete a 54 kDa serine protease that specifically hydrolyzes the receptor-binding domains of *C. difficile* toxins A and B in the intestinal lumen, directly neutralizing their enterotoxicity and inhibiting downstream inflammatory responses (51). Its primary advantage lies in its strong adaptability to the human gastrointestinal environment. The yeast cells can remain viable in the gastric environment at pH 2–3 and transiently colonize the intestine, where they exert their probiotic effects. The acid tolerance of *S. boulardii* is primarily attributed to differences in membrane sterol composition, expression of specific transporters, and increased H^+^-ATPase activity (52). In yogurt, kefir, and cheese, the strain can remain above typical probiotic thresholds when cold storage and compatible starter cultures are used; one yogurt system containing fruit pieces maintained at least 2 × 10^7^ CFU/g after 16 days at 4 °C (53). Yet the same live-cell advantage becomes a limitation under pasteurization, baking, extrusion, or prolonged oxygen exposure. Therefore, live-cell products should define a target viable count at the end of shelf life, not only at inoculation, and should report strain identity, enumeration method, and release kinetics after simulated digestion.

Co-culture studies involving *S. boulardii* and lactic acid bacteria have shown that *S. boulardii* can modulate the environmental pH by metabolizing organic acids such as lactic acid, thereby promoting the growth of lactic acid bacteria. This effect provides a more favorable environment for acid-sensitive strains, including *Lactobacillus casei* and *Bifidobacterium lactis*, resulting in an increase of approximately 1 log unit in the total viable probiotic count in the final product, along with improvements in sensory quality and functional activity (50). Furthermore, co-culture studies reveal a synergistic mechanism: *S. boulardii* modulates the environmental pH by metabolizing organic acids, thereby promoting the growth of acid-sensitive lactic acid bacteria (e.g., *L. casei*), which improves both the sensory quality and functional activity of the final product (54). However, the application of live cells is strictly limited by thermal food processing. Baking (e.g., >180 °C) rapidly depletes viability to <5%. To address this, modern delivery technologies, such as spray-dried microencapsulation using whey protein, are employed to ensure targeted intestinal delivery without compromising the live cells (55, 56). However, excessive yeast metabolism can cause CO_2_ formation, ethanol notes, and texture defects; thus, inoculum ratio, fermentation endpoint pH, storage temperature, and oxygen ingress should be optimized by design-of-experiments rather than reported only descriptively. Microencapsulation with whey protein, alginate, or plant polysaccharides can improve gastrointestinal release and heat tolerance, but scale-up requires quantification of cost, capsule size distribution, sensory roughness, and survival after industrial mixing.

#### 4.4.2 Fermentation biocatalyst

*S. boulardii* can secrete a variety of enzymes, including *β*-glucosidase, protease, and lipase, during fermentation. These enzymes participate in the biotransformation of complex substrates, thereby enhancing the nutritional value and functional properties of foods. The β-glucosidase activity of *S. boulardii* has been reported to be significantly higher than that of other probiotics, resulting in a 3.4-fold increase in the conversion rate of grape skin aglycone-type polyphenols, effectively increasing the concentration of free anthocyanins in fermented blueberry juice and improving the DPPH radical scavenging activity by 42% (57). In fermented lupin, *S. boulardii* induces the coordinated expression of *α*-galactosidase and phytase, leading to significant degradation of antinutritional factors, including oligosaccharides (27.3–82.3%), phytic acid (61.9–67.0%), and alkaloids (25.5–36.7%) (58). In yam-based products, ethanol extraction has been associated with a significant upregulation of various bioactive components and biological activities. In particular, total polyphenol and flavonoid contents increase significantly, accompanied by enhanced antioxidant and immunomodulatory activities (59).

The structure–function relationship of paraprobiotics is heavily reliant on pattern recognition receptor (PRR) engagement. The *S. boulardii* cell wall is uniquely enriched in β-1,3-glucans and highly glycosylated mannoproteins (60). The β-glucan fraction specifically binds to the Dectin-1 receptor on dendritic cells and macrophages, triggering the Syk-CARD9 signaling cascade. This leads to the nuclear translocation of NF-κB and the subsequent upregulation of the anti-inflammatory cytokine IL-10 (46, 61). Mannoproteins in the outer cell wall interact with Dectin-2 on macrophages, further modulating immune responses.

Furthermore, *S. boulardii* can synthesize a range of bioactive metabolites, including short-chain fatty acids (SCFAs), phenyllactic acid, and 2-hydroxyisocaproic acid. These compounds exhibit antimicrobial, anti-inflammatory, and immunomodulatory activities, thereby conferring additional health benefits to fermented foods (62). The application potential of *S. boulardii* can be expanded through genetic engineering. For example, integrating the cHLY gene derived from human breast milk into the genome of *S. boulardii* enables the recombinant strain to continuously secrete lysozyme for up to 48 h under simulated gastric conditions (30 °C, pH 4.5), achieving a yield of 0.8 μg/mL. This recombinant strain effectively inhibited foodborne pathogens, including *Listeria monocytogenes*, and demonstrated significantly greater efficacy compared with the empty vector control strain (63).

#### 4.4.3 Inactivated postbiotic ingredients

With the emergence of the postbiotic concept, non-viable forms of *S. boulardii* have attracted increasing attention. Postbiotics include cellular components, such as cell wall polysaccharides and mannans, as well as metabolites, and offer advantages including high stability, strong safety, and ease of storage (64). Studies have shown that *β*-glucan and mannan in the cell wall of *S. boulardii* exhibit immunomodulatory, anti-inflammatory, and cholesterol-lowering activities, making them suitable for use as functional food ingredients. In particular, heat inactivation (121 °C, 15 min) preserves the structural integrity of mannan in the *S. boulardii* cell wall, enabling it to bind to the Dectin-1 receptor on macrophages, effectively induce IL-10 secretion, and exert significant anti-inflammatory effects in a Caco-2 inflammation model (25). In addition, the incorporation of inactivated yeast powder into whole-wheat bread at a concentration of 1% (w/w) resulted in *γ*-aminobutyric acid (GABA) concentration of 128 μg/mL in the dough and 30 μg/g in the baked bread, while also maintaining strong antioxidant capacity (12%), thereby enhancing the postbiotic properties of the bread (65). Furthermore, heat-inactivated *S. boulardii* increases the expression of tight junction proteins, including occludin and ZO-1, while reducing serum levels of pro-inflammatory cytokines (TNF-*α*, IL-1β, and IL-6) and suppressing their mRNA expression in the colon. Notably, disrupted cells of heat-inactivated *S. boulardii* also modulate gut microbiota composition in dextran sulfate sodium (DSS)-induced dysbiosis (66).

**Figure 3:**
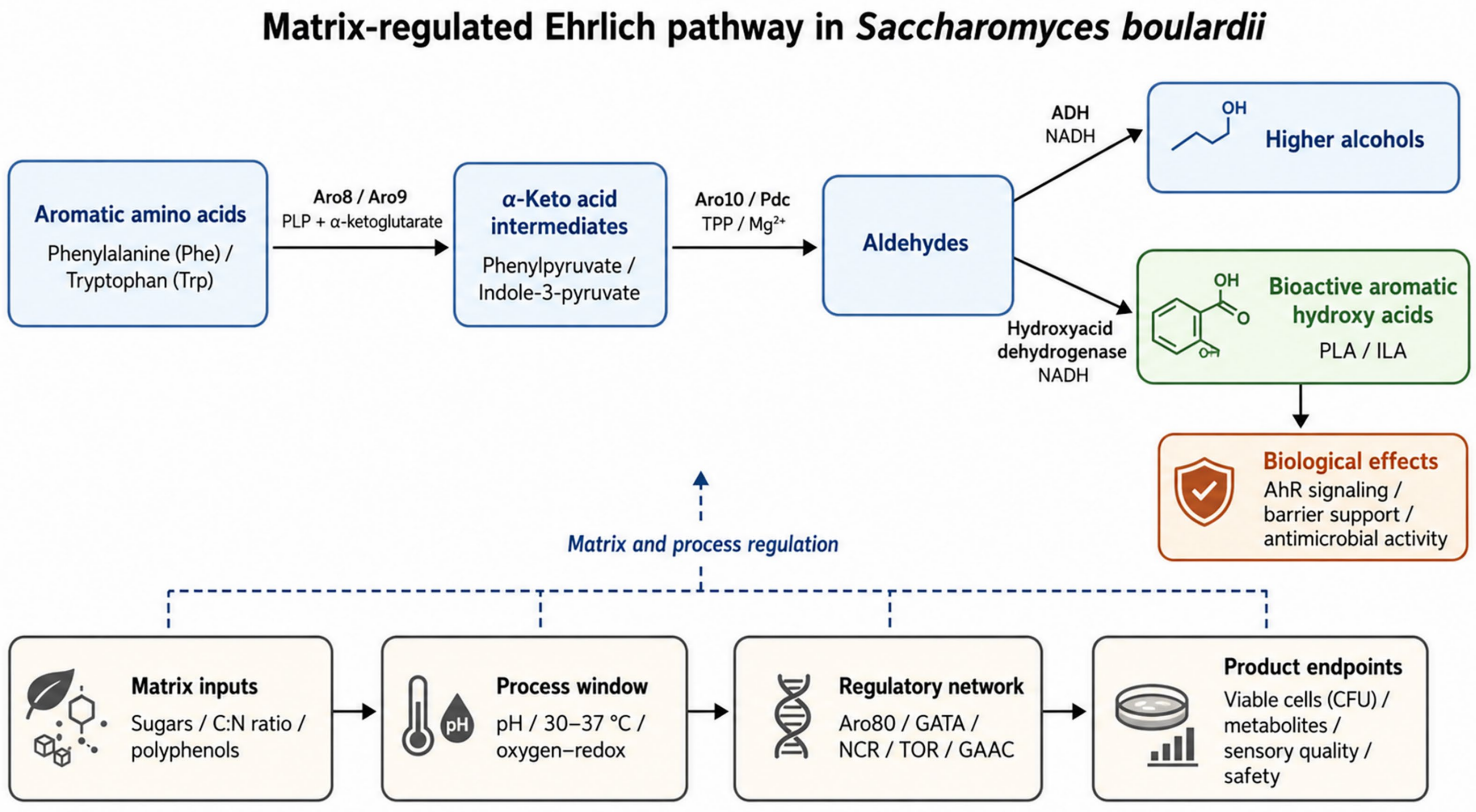
Matrix-regulated Ehrlich pathway in *S. boulardii*. The diagram links aromatic amino acid substrates, key enzymes/cofactors, fermentation parameters, and functional food outcomes.

Beyond enzymatic biotransformation, the therapeutic depth of *S. boulardii* postbiotics depends on defined biosynthetic pathways and on whether food-processing conditions preserve the active molecules. The aromatic amino acid/Ehrlich module (Figure 3) is especially important because it links substrate availability in the food matrix to antimicrobial, epithelial, and sensory endpoints.

Phenyllactic acid (PLA), indole-3-lactic acid (ILA), and related aromatic metabolites are generated from phenylalanine or tryptophan through a three-stage Ehrlich-type pathway. Aromatic aminotransferases (Aro8/Aro9-like enzymes; pyridoxal-5-phosphate dependent) transfer the amino group to α-ketoglutarate, yielding phenylpyruvate or indole-3-pyruvate and glutamate. Thiamine-pyrophosphate/Mg^2+^-dependent decarboxylases (Aro10/Pdc-like enzymes) then generate aromatic aldehydes, while alcohol dehydrogenases, aldehyde dehydrogenases, and aromatic hydroxyacid dehydrogenase-like reactions determine whether flux ends in alcohols, acids, PLA, or ILA. Regulation is controlled by aromatic amino acid availability, nitrogen catabolite repression, TOR/GAAC signaling, Aro80 and GATA factors such as Gln3/Gat1; therefore, carbon source, C/N ratio, oxygen/redox balance, pH, and fermentation time can shift metabolite yield (50). In food matrices, high fermentable sugar and limited oxygen favor NADH-dependent reduction, while phenolics can both inhibit yeast growth and induce β-glucosidase-mediated release of bound antioxidants.

Functional consequences differ by metabolite. ILA can act as an aryl hydrocarbon receptor (AhR)-linked epithelial modulator and may strengthen barrier integrity; PLA is more directly antimicrobial because its lipophilicity enables membrane perturbation and intracellular acidification; and thioredoxin (Trx) is a redox-active extracellular protein that transactivates EGFR/ERK–MAPK signaling, increasing ZO-1 and occludin expression during barrier repair (67). However, Trx and many enzymes are heat-labile, whereas PLA, ILA, and SCFA derivatives are comparatively more compatible with postbiotic or heat-processed products.

The paraprobiotic fraction also requires structure–function control. Heat-killed *S. boulardii* retains the ability to increase tight-junction proteins (occludin and ZO-1) and modulate gut microbiota *in vivo*, but bioactivity depends on the integrity and accessibility of β-glucan/mannan epitopes (68). In food processing applications, production workflows are often constrained by product type and economic considerations. Under such conditions, the use of probiotics in the form of postbiotics as nutritional supplements may confer better probiotic properties. However, sensory attributes and nutritional quality must also be carefully considered (69). For industrial food processing, inactivation should be standardized as a bioprocessing step rather than treated as accidental cell death: the target should be a reproducible combination of viability loss, ligand preservation, metabolite retention, and acceptable sensory impact.

#### 4.4.4 Molecular mechanisms and comparative analysis of *S. boulardii* forms: live cells, paraprobiotics, and postbiotics

The physiological and therapeutic efficacy of *S. boulardii* is highly dependent on its physical state and application form. Recent advances in microbiome research necessitate a clear molecular differentiation among live probiotic cells, paraprobiotics and postbiotics. Each form exhibits distinct structure–function relationships, biochemical mechanisms, and varying stability profiles when subjected to food processing technologies. To systematically differentiate these forms, Table 1 summarizes their key components, molecular mechanisms, stability in food systems, and current clinical evidence strength.

**Table 1:** Comparative analysis of *S. boulardii* forms in food systems herapeutic translation and GRADE-oriented evidence statements.

Application form	Dominant bioactive fraction	Primary molecular mechanisms	Quantitative food-processing implication	Evidence certainty/key limitation
Live Probiotic Cells	Viable yeast cells, active intracellular enzymes, dynamic secretome	54-kDa serine protease and 120-kDa phosphatase neutralize toxins/LPS; live metabolism modulates pH, LAB ecology, and organic acids.	Best for refrigerated dairy and beverages. Examples: yogurt ≥2 × 10^7^ CFU/g after 16 d at 4 °C; ice cream around 6.16 log CFU/g after 120 d. Poor survival in baking/extrusion without encapsulation.	Clinical evidence: moderate for selected diarrhea outcomes; food-matrix directness often low. Limitations: cold chain, dose loss, fungemia risk in vulnerable patients.
Paraprobiotics	Inactivated whole cells, cell wall fragments (*β*-1,3-glucans, mannoproteins, chitin)	Dectin-1/Dectin-2/TLR recognition; Syk-CARD9-NF-κB and IL-10/Treg modulation; processing may expose or mask cell-wall epitopes.	High heat tolerance; suitable for bakery/extrusion when live cells cannot survive. Requires standardized inactivation and epitope-retention assays.	Mechanistic/animal evidence: low-to-moderate; human food trials limited. Limitation: no active secretion or dynamic colonization.
Postbiotics	Secreted enzymes, Phenyllactic acid, Indole-3-lactic acid, Thioredoxin (Trx), SCFAs	Ehrlich-pathway metabolites activate AhR/barrier pathways or disrupt pathogens; Trx transactivates EGFR/ERK–MAPK and increases ZO-1/occludin.	Small organic acids are comparatively heat/pH stable; proteins such as Trx and enzymes are heat-labile. Postbiotic bread reported about 30 microgram/g GABA after baking.	Mechanistic evidence strong but clinical certainty low for purified food-delivered postbiotics. Limitation: potency assays and batch consistency are underdeveloped.

### 4.5 Research progress in food applications

Food applications are evaluated below according to matrix function rather than product category alone. A matrix can act as (i) a protective carrier for live cells, (ii) a fermentation substrate that increases bioactive metabolites, (iii) a sensory system that must mask yeast notes, or (iv) a postbiotic delivery vehicle for heat-stable molecules. Table 2 has been include quantitative endpoint measures where possible, such as viable counts, fold changes in metabolites, antinutritional factor reduction, and sensory or shelf-life effects wherever available.

**Table 2:** Applications of selected probiotic formulations containing *S. boulardii* in foods.

Product type	Product name	Probiotic strain	Main findings	References
Fermented Dairy Products	Yogurt	*L. delbrueckii; S. thermophilus; S. boulardii*	Exhibits five key metabolic pathways, including ascorbate and aldarate metabolism, pentose and glucuronate interconversions, the citrate cycle (TCA cycle), galactose metabolism, and glyoxylate and dicarboxylate metabolism.	(122)
	Cheese	*S. thermophilus; L. delbrueckii*	Adjunct culture improved sensory properties and pathogen control; quantitative sensory descriptors and volatile thresholds remain underreported.	(123)
	Kefir	*S. thermophilus; L. lactis; L. cremoris; L. diacetylactis; L. mesenteroides; S. boulardii*	*S. boulardii* produced more CO_2_ than other yeasts and had favorable taste compatibility; inoculum must be controlled to avoid excessive gas/ethanol notes.	(124)
	Ice cream	*L. delbrueckii; S. thermophilus; S. boulardii*	Maintained probiotic viability (6.16 log CFU/g after 120 d), improved product stability, reduced hardness, improved melting behavior, and increased volatile diversity.	(125)
Plant-Based Foods	Fermented soybean meal	*B. velezensis; E. faecium; S. boulardii*	Mixed fermentation increased small peptides/TCA-soluble protein; reduced oligosaccharides (27.3–82.3%), phytic acid (61.9–67.0%), and alkaloids (25.5–36.7%).	(126)
	Fermented Chinese yam	*S. boulardii*	Fermentation lowered polysaccharide molecular weight and enhanced antioxidant activity; bioaccessibility after digestion should be reported with confidence intervals.	(75)
	Fermented apple puree	*S. boulardii*	Fermented apple matrix increased phenolics/quercetin and antioxidant activity; sugar/phenolic balance should be linked to yeast survival and sensory acidity.	(12)
Non-Dairy Fermented Beverages	Beer	*S. boulardii*	Ferments at low temperatures (2 °C) and is applicable in non-alcoholic and low-alcohol products.	(13)
	Vegetable juice	*L. plantarum; L. rhamnosus; L. paracasei; S. boulardii*	Prebiotic activity observed, but direct health evidence is indirect; viability, pH, sugar depletion, and shelf-life stability should be quantified.	(127)
	Fermented tea	*L. plantarum; S. boulardii*	Reduced flavonoid glycosides and altered tea metabolome; β-glucosidase activity links matrix biotransformation to antioxidant/sensory endpoints.	(128)
FSMP	Oral supplements	*S. boulardii*	Oral supplement evidence improves microbiota evenness but has low food-matrix directness; should not be treated as equivalent to a food vehicle.	(129)
	Commercial food supplements	*L. acidophilus; L. casei; B. bifidum; S. boulardii*	Antibacterial/antifungal activity documented; product-specific viable dose and strain identity should be declared.	(32)
High-Temperature Processed Foods	Postbiotic-enriched bread	*L. plantarum; S. boulardii*	Increases γ-aminobutyric acid (GABA) content and exhibits strong antioxidant capacity.	(130)
	Probiotic cereal bars	*S. boulardii*	Acacia gum/coating improves thermal stability in coated cereal products; needs quantitative CFU retention after industrial baking/extrusion.	(131)
Edible Food Packaging Materials	Alginate-based edible films	*S. boulardii*	Increases the concentration of bioactive compounds and antioxidant activity.	(18)
	Edible meat coating	*S. boulardii*	Postbiotic coating reduced oxidation products and delayed microbial growth in lamb meat; regulatory labeling and sensory effect require testing.	(19)

#### 4.5.1 Fermented dairy products

Dairy matrices remain the most mature delivery vehicles because buffering capacity, proteins, fat, and refrigerated distribution protect yeast survival. In yogurt, *S. boulardii* can stimulate LAB and maintain probiotic counts above 10^6^ CFU/mL at the end of shelf life, an issue emphasized in dairy-focused reviews (50). Sarwar et al. (70) demonstrated that yogurt produced through symbiotic fermentation with *S. boulardii* and LAB exhibited a higher solid–liquid balance value, a significantly greater diversity of volatile compounds, and a markedly reduced fat content. In goat milk yogurt, *S. boulardii* maintained high viability over a four-week storage period without causing significant changes in taste compared with conventional yogurt, thereby preserving its characteristic flavor. Importantly, LAB survival was significantly higher in the presence of *S. boulardii* than in the control group, indicating that the yeast enhances LAB viability (71). In recent years, *S. boulardii* has also been widely used in frozen dairy products such as ice cream. Sarwar et al. (72) developed a probiotic ice cream using a co-fermentation system of *S. boulardii* CNCM I-745 together with *Lactobacillus* and *Streptococcus* strains. After 120 days of storage, the viable probiotic count remained at 6.16 log CFU/g. Co-fermentation with the probiotic yeast and LAB significantly reduced ice cream hardness, thereby improving its texture. Measurements of particle size, zeta potential, and multiple light scattering further indicated enhanced melting behavior and product stability. Nevertheless, sensory evidence remains uneven: many studies report overall acceptability but do not provide quantitative volatile thresholds, consumer segmentation, or off-flavor drivers such as ethanol and CO_2_. This review therefore treats dairy as technologically promising but still requiring standardized sensory panels and volatile-metabolomics endpoints.

#### 4.5.2 Plant-based foods

In response to the needs of individuals with lactose intolerance and vegetarian diets, *S. boulardii* has been successfully incorporated into plant-based matrices, such as soybean, oat, apple, and nut-based products. During mixed fermentation of soybean flour with *Bacillus velezensis*, *Enterococcus faecium*, and *S. boulardii* at 46 °C for 6 days, the small peptide content increased approximately 11-fold, while antinutritional factors such as urease activity and phytic acid, as well as contaminating microorganisms, were reduced. These results indicate that one-step mixed fermentation driven by protease activity can effectively enhance the nutritional value of soybean flour (73). During fermentation of lentil and red bean sprouts, *S. boulardii* remained viable throughout simulated digestion and exhibited significant antimicrobial activity against molds and *E. coli*. Extract analyses further revealed that fermentation significantly increased the content of antioxidant compounds, such as phenolics, with higher extraction efficiency observed in red bean sprouts, where the main components were identified as catechin and quercetin glycosides (74). In addition, *S. boulardii* has been used as a starter culture in the fermentation of Chinese yams to evaluate the probiotic properties of the resulting fermented products. The results demonstrated that fermentation with *S. boulardii* produced Chinese yam polysaccharides (FCYP) with lower molecular weight and enhanced antioxidant activity, along with a mild radioprotective effect. Furthermore, the antioxidant compounds in FCYP remained active throughout gastrointestinal digestion, indicating good stability and bioaccessibility (75).

#### 4.5.3 Bakery and snack foods

Baking temperatures of 180–220 °C far exceed the tolerance limit of *S. boulardii*, with viability dropping to <5% after 1 h at 52 °C. Therefore, common approaches include encapsulating *S. boulardii* in microcapsules or incorporating postbiotics into foods, both of which help preserve its probiotic properties. Studies have demonstrated that single- and double-layer microcapsules containing *S. boulardii*, *Lactobacillus acidophilus*, and *Bifidobacterium bifidum*, prepared through spray drying and cooling, can be incorporated into cakes. After baking at 200 °C for 20 min, the viable counts of *S. boulardii* and *L. acidophilus* in double-layer microcapsules reached 2.9 log CFU/g, with survival rates of 67.4 and 70.7%, respectively. Furthermore, no significant differences in texture characteristics were observed between cakes containing *S. boulardii* and those without *S. boulardii*. (76). In functional probiotic breakfast products such as corn flakes supplemented with *S. boulardii*, the use of acacia gum as a coating material has been shown to enhance heat tolerance. After heat treatment at 80 °C and subsequent storage at 30 ± 2 °C for 90 days, the survival rate of *S. boulardii* remained as high as 88.3%. In addition, *S. boulardii* exhibited strong resistance to gastric acid and pepsin, as well as moderate resistance to pancreatic juice (77).

#### 4.5.4 Non-dairy fermented beverages

In craft beer, wine, and mead, *S. boulardii* has been shown to maintain viability while improving sensory and functional properties, including the production of bioactive components such as phenolic compounds and antioxidants (78). *In vitro* gastrointestinal digestion models have indicated that black carrot juice fermented with *S. boulardii* exhibited high antioxidant activity associated with increased total phenolic content, while fermented red beet juice showed higher bioaccessibility of antioxidant activity (79). A study on green tea beverages co-fermented with *S. boulardii* and *Lactiplantibacillus plantarum* revealed that co-inoculation significantly improved LAB survival. Moreover, co-culture enhanced the production of aromatic compounds. Compared with single-culture fermentation, co-fermentation significantly increased the concentrations of methyl salicylate, methionol, and 2-phenylethanol (80). When *S. boulardii* was added as a starter culture in barley wort fermentation, the concentrations of oligosaccharides, including maltotriose, maltotetraose, maltopentaose, maltohexaose, and maltoheptaose, increased significantly. Oligosaccharide profile analysis showed that the fermented beverage contained higher levels of prebiotic oligosaccharides than unfermented barley malt and its extracts (17). Overall, beverages fermented with *S. boulardii* exhibit enhanced functional properties and improved sensory quality, aligning with the growing demand for probiotic and functional beverages.

#### 4.5.5 FSMP

*S. boulardii* CNCM I-745 has been included in the European Union list of foods for special medical purposes (81). *S. boulardii* is more effective than bacterial probiotics in preventing antibiotic-associated diarrhea (AAD) and pediatric acute gastroenteritis (PAGE), and its efficacy in controlling diarrhea exceeds that of conventional antidiarrheal drugs or oral rehydration therapy (82). Patients receiving oral administration of *S. boulardii* showed a significant reduction in gastrointestinal symptoms and greater evenness in gut microbial diversity. After treatment, the abundance of *Bacteroides* and *Clostridium* decreased, along with a reduction in *Helicobacter pylori* levels in the patients’ intestines, resulting in alleviation of associated symptoms (83). For pediatric patients with irritable bowel syndrome (IBS), an FSMP specifically developed for children using 0.5% galacto-oligosaccharides and 0.3% glutamine as a synergistic matrix significantly improved yeast survival under simulated pediatric gastrointestinal conditions and reduced inflammatory cytokines such as TNF-*α* and IL-8 (63). In preterm neonates, oral administration of *S. boulardii* CNCM I-745 has been associated with significantly improved growth rates and feeding tolerance. Prophylactic supplementation with *S. boulardii* was also found to promote weight gain and improve feeding tolerance (84).

#### 4.5.6 Food packaging materials

In recent years, technological advances have led to the development of a new generation of food packaging systems that incorporate functional ingredients such as prebiotics, probiotics, bioactive peptides, vitamins, and other bioactive compounds, thereby contributing to improved consumer health (85). Studies have shown that alginate-based bioactive edible films supplemented with mangaba fruit pulp and *S. boulardii* can serve as effective food packaging materials. The probiotic culture can effectively colonize the film and maintain high viability even after prolonged storage (21 d). Moreover, incorporation of bioactive compounds such as carotenoids, vitamin C, and phenolic compounds enhances the antioxidant and UV-protective properties of the film (18). An edible coating developed using *S. boulardii* seed mucilage and *S. boulardii* has been effectively used for preserving lamb meat. This coating significantly reduced the growth of microorganisms, including *Salmonella*, *E. coli*, *Bacillus cereus*, *Staphylococcus aureus*, and *Listeria monocytogenes*, and extended the shelf life of the meat to more than 10 days. At the same time, key quality attributes such as moisture content, pH, and hardness were better maintained (19).

#### 4.5.7 Assessment of the feasibility of *S. boulardii* in different food matrices

Across matrices, the main feasibility gradient is clear: refrigerated dairy and acidic beverages are most suitable for live-cell delivery; plant matrices are valuable for biotransformation of phenolics, peptides, and antinutritional factors; bakery, extruded, and ready-to-eat foods are better suited to paraprobiotic or postbiotic formats unless protective encapsulation is validated; and edible films/coatings are promising for preservation but require migration, dose uniformity, and sensory testing. While *S. boulardii* generally exhibits robust survival compared to bacterial probiotics, existing literature presents contradictory findings regarding its viability in various food matrices. For instance, while some studies report stability in dairy yogurts, others demonstrate a significant decline in viability during shelf life in acidified fruit juices. This discrepancy is likely not due to the strain itself, but rather to the complex interactions between the matrix’s buffering capacity, pH, and the availability of specific micronutrients. Quantitative comparison is still limited because studies use different strains, inocula, storage durations, and enumeration methods. Future research must move beyond reporting survival percentages and systematically compare the matrix effect—specifically how the structural components (e.g., protein-polysaccharide complexes) provide protective barriers—versus the inhibitory effects of organic acids and redox potential in different beverages.

### 4.6 Evidence for health benefits

#### 4.6.1 Acute infectious diarrhea

In acute diarrhea caused by rotavirus or norovirus, *S. boulardii* counteracts pathogen-induced toxins by secreting a 54 kDa protease while simultaneously enhancing intestinal barrier integrity (86). Studies indicate that incorporating *S. boulardii* into yogurt (≥ 10^6^ CFU/g) maintains viability under gastric conditions and supports metabolic activity in milk through lactose-derived substrates, such as lactic acid and galactose, thereby enhancing its antidiarrheal potential. For example, Ragavan et al. (87) reported a pediatric intervention for acute diarrhea using lactose-free milk supplemented with *S. boulardii* (1,306 children, including 651 in the *S. boulardii* group and 655 in the control group). The duration of diarrhea was significantly reduced by an average of 24 h, and body weight increased in the treated group. The GRADE certainty is judged as moderate for short-term symptom reduction in children because RCTs exist, but it is downgraded for heterogeneity, variable background rehydration therapy, and limited direct use of food matrices. Food developers should therefore avoid extrapolating capsule efficacy to dairy or beverage products unless the final product delivers the same strain and viable dose at consumption.

#### 4.6.2 AAD

The occurrence of AAD is closely associated with gut microbiota dysbiosis induced by antibiotic therapy. Studies have demonstrated that oral administration of *S. boulardii* initiated 48 h after the start of antibiotic treatment significantly reduced the incidence of AAD from 9 to 1.4%. Prophylactic administration further reduced the risk of AAD in hospitalized patients, and no severe AAD cases were reported (88). Infants often develop AAD following antibiotic treatment. In a 7-day intervention study (*n =* 110), infants who consumed 50 g of yogurt containing 10 billion CFU of *S. boulardii* as a complementary food reduced the incidence of diarrhea from 20 to 10%. Microbiota diversity analysis of fecal samples indicated improved gut microbial diversity and community structure (89). This inconsistency leads to moderate-to-low certainty depending on population and baseline risk. The Table 3 therefore distinguishes pediatric/high-risk contexts from unselected hospitalized adults and explicitly notes that food-matrix RCTs are scarce (90).

**Table 3:** A GRADE-based certainty assessment of the clinical evidence for *S. boulardii* interventions and their translation into food matrices.

Disease/Indication	Target population	Common dosage regimen	Outcome and GRADE-oriented certainty	Main effect/evidence strength
Acute gastroenteritis	Infants /children	250 mg/day (may be divided), up to 5 days	Reduced diarrhea duration and improved symptoms; generally well tolerated. Some concerns; moderate certainty.	Modestly shortens diarrhea duration; good safety profile (98)
AAD	Infants / children	250 mg/day, starting with antibiotics for approx. 2 weeks	Reduced AAD incidence in several studies; magnitude varies by age/risk. Moderate-to-low certainty.	Significantly reduces AAD incidence; well tolerated. In children ≤1 year, AAD risk reduced by 52% (132)
	Adults	500 mg/day (divided into two doses) for 21 days	Large RCT found no benefit; earlier meta-analyses suggest benefit in selected contexts. Low-to-moderate certainty due to inconsistency.	Standard prophylactic regimen; further studies needed to confirm efficacy (99)
IBS	Adults	750 mg/day for 6 weeks	Possible symptom/cytokine improvement but heterogeneity high. Low certainty.	Clinically effective; improves cytokine profile, histological features, and quality of life compared with placebo (100)
Traveler’s diarrhea (prevention)	Healthy adults	High dose (e.g., 250 mg vs. 1,000 mg)	Dose–response trend at higher intake. Low-to-moderate certainty.	Dose–response relationship observed: higher dose (1,000 mg/day) shows better protection than lower dose (250 mg/day) (101)
*C. difficile* infection prevention	Hospitalized adults	250 mg twice daily	Toxin mechanisms strong but clinical prevention evidence inconsistent. Low certainty.	Insufficient evidence of benefit; routine use not recommended (133)

The dosage unit (mg) varies depending on the product, and the number of viable cells (CFU) per 1 mg is not constant. Clinical efficacy is also influenced by factors such as the target population and the specific disease.

#### 4.6.3 Inflammatory bowel disease (IBD) and IBS

In addition, *β*-glucan in the inner layer of the yeast cell wall binds to the Dectin-1 receptor on macrophages and DCs, promoting the secretion of immunomodulatory cytokines such as TNF-α and IL-6. Mannoproteins in the outer cell wall interact with Dectin-2 on macrophages, thereby promoting cytokine secretion and modulating immune responses. This regulation is achieved through anti-inflammatory cytokines such as IL-10 (61). A randomized double-blind clinical trial evaluated the effects of *S. boulardii* in patients with irritable bowel syndrome (*n =* 179). After daily administration of capsules containing 500 mg of live *S. boulardii* granules for 8 weeks, followed by a 3-week washout period, the proportion of symptom responders was significantly higher in the treatment group compared with the placebo group (63% vs. 47%) (91). Gu et al. (92) found that *S. boulardii* fermentation supernatant (SbS) activates the EGFR pathway by enhancing the release of heparin-binding epidermal growth factor (HB-EGF), thereby upregulating SERT expression. Treatment with an EGFR kinase inhibitor or transfection with HB-EGF siRNA blocked the SbS-induced upregulation of SERT in cells. *In vivo, mice* treated with SbS exhibited reduced intestinal motility, thereby alleviating IBS symptoms.

#### 4.6.4 Immune and metabolic syndrome

*In vivo* studies have demonstrated that animals supplemented with fermented whey exhibited modulated immune responses, including significantly elevated IgA levels, suggesting enhanced mucosal immunity (93). Studies have further demonstrated that *S. boulardii* inhibits the proliferation of T helper 17 (Th17) cells and type 3 innate lymphoid cells (ILC3s), while simultaneously reducing intestinal permeability, promoting the expression tight junction proteins, modulating the gut microbiota, and lowering the levels of inflammatory cytokines in rats, thereby enhancing bone and joint integrity and improving immune function (22). Oral administration of *S. boulardii* has also been shown to alter gut microbiota composition in mice, promote the growth of *Clostridium* species, and increase microbial diversity. In addition, the administration of *S. boulardii* affected host energy metabolism and effectively reduced blood urea and fructose levels, indicating that this probiotic yeast can exert health-modulating effects in mice (94).

#### 4.6.5 Antitumor and antioxidant effects

Recent studies have demonstrated that *S. boulardii*, similar to certain probiotic microorganisms, may be used for cancer prevention or as an adjuvant in anticancer chemotherapy by modulating the gut microbiota and host immune responses (95). Metabolites produced by *S. boulardii*, including β-glucan, phenyllactic acid, and vanillic acid, exhibit significant antioxidant and antitumor activities. These compounds can induce apoptosis in colon cancer cells by inhibiting the EGFR/Akt pathway (96). Qin et al. (43) reported that oral administration of SbS effectively alleviated intestinal inflammation and reversed DSS-induced downregulation of EGFR activation in a colitis model. In addition, Trx produced during fermentation exhibited multiple anti-inflammatory effects, including attenuation of inflammation, protection of the intestinal barrier, inhibition of apoptosis, and reduction of oxidative stress. Oral administration of *S. boulardii* has also been shown to increase C-peptide secretion and effectively reduce blood glucose levels in diabetic animal models. Furthermore, *S. boulardii* enhanced renal antioxidant defenses, restored serotonin and dopamine concentrations, and activated the vasodilatory and antifibrotic axes of the renin-angiotensin system (RAS), demonstrating that long-term administration of *S. boulardii* exerts hypoglycemic and antioxidant effects in mice (97).

#### 4.6.6 Dose–response relationship in clinical treatment

In pediatric acute diarrhea, a higher single dose is not always superior. Studies indicate that 250 mg/day is effective and safe for reducing disease duration and alleviating symptoms. Furthermore, animal studies suggest that moderate to low doses (100–500 million CFU/g) are more effective than high doses (1 billion CFU/g) in modulating the gut microbiota (98). This implies a potential “plateau effect” in functional regulation, rather than a simple linear relationship. For the prevention of AAD, 250 mg/day (administered once or twice daily) is an effective and commonly used dosage in children, whereas 500 mg/day (administered twice daily) is typically utilized in adult studies (99). Despite these dosage variations, current evidence supports the efficacy of *S. boulardii* at these specific doses for AAD prevention. Regarding adult IBS treatment and traveler’s diarrhea prevention, evidence supporting higher doses is more pronounced. In IBS treatment studies, the effective dose was 750 mg/day (100). For traveler’s diarrhea prevention, a large-scale study demonstrated that a high dose (1,000 mg/day) provided superior protective efficacy compared to a low dose (250 mg/day) (101), representing the most compelling direct evidence for a dose–response relationship.

Currently, there is a lack of unified and precise conclusions regarding the dose–response relationship of *S. boulardii* clinical practice. A synthesis of existing evidence suggests that its efficacy is highly dependent on factors such as strain specificity, disease type, patient age, treatment duration, and combination therapy (Table 3). Treatment regimens should be tailored to individual variability, and pediatric dosing may require age-adjusted modifications (e.g., halving the dose for infants under 1 year old). Additionally, combining *S. boulardii* with *Bifidobacterium* or using it as an adjuvant therapy alongside mesalamine may confer additional benefits. Future dosage studies should consider these complex clinical application scenarios. A critical barrier in the current literature is the unjustified assumption of “class effect” regarding *S. boulardii*. Clinical outcomes are frequently attributed to the species as a whole, ignoring the significant inter-strain variance in genomic plasticity and metabolite secretion profiles. Critically, we identify that the efficacy of *S. boulardii* is non-linear; the common clinical “standard dose” often fails to account for the temporal requirement for colonization resistance.

Dose–response interpretation should combine viable dose, matrix protection, and timing rather than nominal mass alone. A dose expressed as mg is not comparable across products unless the viable count, strain identity, and delivery system are specified. In food systems, the effective dose may be altered by gastric buffering, fat/protein protection, encapsulation release, and microbial interactions. Therefore, future RCT tables should report CFU at manufacture, CFU at consumption, survival after simulated digestion, and actual intake during the intervention.

Overall, the clinical synthesis now supports a more cautious claim structure: strong biological plausibility, moderate evidence for selected diarrheal outcomes, lower certainty for IBS/IBD/metabolic outcomes, and very limited direct evidence for postbiotic or engineered *S. boulardii* delivered in foods. This critical appraisal addresses the need for quantitative synthesis and avoids overstating food-health claims from non-food trials.

## 5 Regulatory status and industrial challenges

### 5.1 Current progress of regulatory systems in major global countries

Since *S. boulardii* was introduced as a prescription drug in France in the 1990s, its transition to food applications has been accompanied by evolving regulatory frameworks and persistent regional differences (102). The United States and the European Union, as the largest functional food markets, adopt fragmented regulatory approaches regarding the classification, market access pathways, and permitted health claims for the same strain. This divergence reflects that probiotic yeasts remain an emerging category within regulatory science.

The U. S. Food and Drug Administration (FDA) classifies *S. boulardii* as a variant of *S. cerevisiae*, allowing its entry into the dietary supplement market via the Generally Recognized as Safe (GRAS) pathway or through a New Dietary Ingredient (NDI) notification (103). However, when the microorganism is used in a novel application or combined with new microbial-derived ingredients, its GRAS status may be reassessed under established scientific procedures (FDA, 2021). GRAS status for a specific application can be obtained through the FDA notification process, resulting in a “no objection” letter issued by the agency. In 2017, Biocodex received the first “no objection” letter for *S. boulardii* CNCM I-745 in a lyophilized formulation, with a maximum daily dose of 1 × 10^10^ CFU, applicable to populations ranging from infants to older adults. However, permitted functional descriptions were limited to the “structure/function” claim category. Statements implying relief of diarrhea or protection against antibiotic-associated conditions were classified as disease claims and were therefore not allowed (104).

The European Union classifies *S. boulardii* as a microbial-derived food, although microbial cultures are not explicitly defined in EU legislation and are instead regulated as basic food ingredients (105). The Qualified Presumption of Safety (QPS) is a generic risk assessment framework used by the European Food Safety Authority (EFSA) for notified biological agents to streamline safety evaluation across different scientific panels and units. As a biological agent, *S. boulardii* must strictly adhere to the relevant QPS requirements (106). According to the QPS list, yeasts are generally considered less pathogenic compared with other microbial groups. However, they can cause diseases under certain growth conditions. Therefore, applicants are still required to provide toxicological assessment and data on fungal antimicrobial resistance as part of the notification process to demonstrate safety (107).

In emerging markets such as China, the regulatory framework for FSMP and probiotic ingredients is strictly overseen by authorities including the State Administration for Market Regulation (SAMR) and the National Health Commission (NHC). Under current global regulatory frameworks and Codex standards, which inform local guidelines, probiotic starter cultures must typically maintain a minimum viable microbial count of 10^7^ CFU/g, and 10^6^ CFU/g for microbes specifically claimed on labels throughout their shelf life (108).

While conventional *S. boulardii* are widely applied, navigating the approval process for novel or modified strains presents significant challenges (109). A major bottleneck is the discrepancy in global regulations and the rigorous safety assessments required to prevent opportunistic pathogenesis; regulatory bodies require extensive proof that new strains will not undergo harmful genetic mutations or cause infections in immunocompromised populations under specific conditions (110). Given the increasing interest in novel biological agents, regulatory support for *S. boulardii* remains limited across different countries, and the review process is stringent. Implementing scientific procedures to assess the safety of novel microbial applications is essential for manufacturers of yeast-based preparations and food producers to ensure product safety.

### 5.2 Technical bottlenecks and safety considerations

At the technical level, the industrial application of *S. boulardii* is constrained by two main bottlenecks: the stability of its probiotic properties and compliance with genetic modification requirements. Although *S. boulardii* can tolerate low pH and relatively high temperatures, viable cells are rapidly inactivated under the processing and sterilization conditions commonly required in food manufacturing, which are dictated by standard food processing technologies (111). To address this limitation, various strategies have been explored to protect probiotic viability, including post-drying microencapsulation (55) and high-pressure processing (112). These approaches can improve microbial survival and stress resistance of probiotics during processing while preserving the nutritional and sensory properties of foods, thereby enhancing food safety and shelf life. Live-cell products require cold-chain control, validated end-of-shelf-life enumeration, strain authentication, and protection against heat, oxygen, osmotic stress, and low water activity. Microencapsulation, spray/freeze drying, high-pressure processing, and protective matrices can improve survival, but they add costs, may alter mouthfeel, and can reduce metabolite release if wall materials are too resistant. Postbiotic approaches improve thermal robustness but require standardization of inactivation, molecular fingerprints, and potency assays.

Sensory scalability remains underdeveloped. Yeast-driven CO_2_, ethanol, higher alcohols, aldehydes, and sulfur-like notes can be desirable in fermented beverages but undesirable in yogurt, FSMP, and pediatric products. Quantitative sensory data should therefore be linked to volatile metabolomics, inoculum level, sugar consumption, acidification kinetics, and storage. For dairy systems, co-culture with *S. thermophilus*, *L. delbrueckii*, *L. casei*, or *Bifidobacterium* strains can balance acidity and aroma, but consumer panels and descriptive analysis are still needed to define acceptance thresholds (113).

Strain engineering offers a forward-looking route for programmable functions, including lysozyme, IL-10, antimicrobial peptides, vaccine antigens, or metabolic payloads (26). Although these genetic engineering tools are now widely available and validation studies involving engineered *S. boulardii* are increasing, clinical trials remain limited, including human studies and applications of modified *S. boulardii* strains as food ingredients. While evidence indicates that engineered probiotic yeasts can produce metabolites targeting specific conditions and deliver them to the intestine, most studies lack translational validation and clinical application (114). Therefore, the regulation of genetically modified probiotics remains an urgent issue that needs to be addressed. Current regulatory frameworks for genetically modified probiotics are highly diverse and lack harmonization (115, 116). For food use, however, engineering must be evaluated through food-grade selection markers, genome stability, off-target edits, plasmid loss, horizontal gene transfer risk, kill-switch performance, manufacturing containment, and regulatory classification. Engineered *S. boulardii* should therefore be discussed as a future platform rather than as a near-term ingredient for conventional foods.

Although genetically engineered probiotics offer programmable therapeutics through technologies like CRISPR-Cas9 editing and genetic circuits, their clinical translation is hindered by severe bottlenecks, including transient colonization and significant intra- and inter-subject microbiome variability (117). Furthermore, introducing genetically modified microorganisms into the human gut poses critical biosafety and ecological risks. A primary ecological concern in microbiome engineering is the control of horizontal gene transfer, where modified traits or antibiotic resistance genes could inadvertently spread to the native microbiota. To mitigate these biocontainment risks, synthetic “kill switches”–such as toxin–antitoxin modules or quorum-sensing circuits—are currently being designed to limit the survival and replication of engineered strains once they exit the host or target niche (118). Ultimately, the clinical adoption of these living therapeutics relies heavily on proving long-term safety and establishing clearly defined ethical and regulatory pathways.

A practical scale-up framework should include: upstream fermentation yield and oxygen control; downstream drying or inactivation yield; protection of enzymatic and metabolite activity; package oxygen and moisture barriers; compatibility with pasteurization or aseptic filling; and batch-release tests for identity, potency, contaminants, and stability. Quality-by-design and life-cycle assessment are needed because the most biologically active format may not be the most scalable or sustainable format.

Safety claims should be stratified by population. *S. boulardii* is generally well tolerated in healthy adults and many pediatric contexts, but fungemia and sepsis have been reported in severely ill, immunocompromised, catheterized, or very young patients (119, 120). For FSMP and clinical nutrition, product labels and trial protocols should define contraindications, viable dose, strain identity, manufacturing controls, and monitoring of adverse events (121). For postbiotics, safety assessment should additionally include residual viable cells, endotoxin-like activity from co-cultures, allergenicity, and batch-to-batch molecular consistency.

## 6 Conclusions and future perspectives

*S. boulardii* is currently the most well-documented probiotic yeast with promising applications in functional foods and the management of certain gastrointestinal disorders. The available evidence, primarily from *in vitro* and animal studies along with a number of clinical trials, indicates that *S. boulardii* may alleviate diarrhea, irritable bowel syndrome, and immune dysfunction, partly through modulation of the gut microbiota. Human studies have demonstrated that *S. boulardii* shows overall efficacy in the treatment of gastrointestinal disorders; however, its specific therapeutic effects are significantly influenced by the strain, actual dosage, and the food matrix in which it is delivered. Therefore, individual patient variability should still be taken into account when designing treatment regimens. *S. boulardii* can be applied in food systems as live cells, fermentation biocatalysts, or postbiotics. Postbiotics, in particular, offer improved stability and are increasingly used in processed foods such as baked goods and extruded snacks. Nevertheless, the translation of these findings into industrial applications faces technical bottlenecks, including poor viability under thermal processing and high production costs associated with microencapsulation. Furthermore, safety considerations—especially for immunocompromised populations—and the lack of harmonized regulatory frameworks remain unresolved challenges. The analysis shows that its value derives from interacting mechanisms: live-cell enzyme secretion and ecological modulation, cell-wall PRR signaling, aromatic amino acid metabolites produced through matrix-regulated pathways, and heat-stable postbiotic fractions. However, the strength of evidence differs substantially by outcome. Selected diarrheal indications have the strongest clinical support, whereas food-matrix RCTs, purified postbiotic trials, engineered strain applications, and quantitative sensory-scalability studies remain insufficient. Future research should not only focus on innovative application strategies for this product but also emphasize *in vivo* validation of *S. boulardii* incorporated into food matrices within the human digestive and immune systems. A key priority is the mechanistic and theoretical validation of metabolites produced by the probiotic, which would provide stronger evidence for its roles in regulating gut microbiota, generating bioactive compounds, and modulating key physiological indicators. Furthermore, ensuring that adequate concentrations of *S. boulardii* or its metabolites reach target sites in the gastrointestinal tract is essential, and future improvements in delivery systems may be required to enhance therapeutic efficacy. Safety remains a paramount concern. Microbial risk assessment should be rigorously applied in all contexts. The safety evaluation of probiotic yeasts in foods is still in its early stages, and harmonized standards based on robust clinical evidence urgently need to be established by relevant regulatory authorities. It is foreseeable that, as *S. boulardii* gains wider approval as a novel food ingredient, this probiotic yeast will continue to expand its presence in the functional food sector, offering new microbial solutions for food innovation.

## Data Availability

The original contributions presented in the study are included in the article/supplementary material, further inquiries can be directed to the corresponding authors.
